# Navigating Discussions around Alzheimer's Disease and Related Dementias: Preliminary Findings from Focus Groups with Black/African American Mid‐Life and Older Adults in South Carolina

**DOI:** 10.1002/alz70858_105547

**Published:** 2025-12-26

**Authors:** Lena Simon, Abigail Stephan, Angie L Sardina, Jeffery Meadows, Marsha Hampton, Lesley A. Ross, Alyssa A. Gamaldo

**Affiliations:** ^1^ Clemson University, Institute for Engaged Aging, Seneca, SC, USA; ^2^ Clemson University, Clemson, SC, USA

## Abstract

**Background:**

The prevalence of Alzheimer's disease and related dementias (ADRD) in South Carolinians is expected to increase by over 26% between 2020‐2025, making South Carolina a priority state for ADRD‐related health research. The effects of ADRD impact both individuals experiencing cognitive decline and their families and communities. Family members and close friends are often involved in healthcare decisions; this may be exceedingly true for minoritized populations, such as Black/African American families, who can have limited access to resources but abundant cultural capital. Despite this, multigenerational family processes are rarely considered in minority aging research. To address this challenge, we conducted an investigation to identify beliefs, expectations, and relevant factors in families’ discussions and decision‐ making around ADRD‐related topics.

**Method:**

Our sample included 40 South Carolinians (Mage=70.25, SD=10.00, range=45‐89 years, 82.5% Women, 97.5% Black/African American) from two churches. We employed a mixed methods approach that emphasized the qualitative strand. Participants engaged in a facilitated focus group discussion with family members/fictive kin, and independently responded to quantitative measures at three time points (pre‐focus group, one‐week post‐focus group, and one month post‐focus group) via an online survey. Preliminary analyses included thematic analysis of focus group responses and descriptive statistics for ADRD knowledge and perceptions from our one week pre assessments.

**Result:**

Findings from the pre‐survey demonstrate that 85% of participants believed the focus groups will be helpful for their family and 77.1% felt they were very likely to engage in ADRD conversations with their family. Three notable themes were identified from the focus groups: 1) decisions on caregiving designated to one family member; 2) middle class struggle with medical insurance; and 3) stigma exists pertaining to ADRD caregiving needs. Another meaningful observation was that conceptions of “family” in relation to ADRD discussions, decision making, and support might extend beyond standard relational ties of blood and marriage.

**Conclusion:**

This exploratory analysis revealed that focus groups appear to be an acceptable method for promoting discussions and ADRD knowledge/awareness among mid‐life and older adult Black/African American South Carolinians and collecting meaningful information about how these conversations are approached within families, friend groups, and communities.

